# Muscular and Prefrontal Cortex Activity during Dual-Task Performing in Young Adults

**DOI:** 10.3390/ejihpe13040055

**Published:** 2023-03-31

**Authors:** Marina Saraiva, Maria António Castro, João Paulo Vilas-Boas

**Affiliations:** 1 RoboCorp Laboratory, i2A, Polytechnic Institute of Coimbra, 3046-854 Coimbra, Portugal; 2 Faculty of Sports and CIAFEL, The University of Porto, 4200-450 Porto, Portugal; 3 Centre for Mechanical Engineering, Materials and Processes, CEMMPRE, University of Coimbra, 3030-788 Coimbra, Portugal; 4 Sector of Physiotherapy, School of Health Sciences, Polytechnic Institute of Leiria, 2411-901 Leiria, Portugal; 5 LABIOMEP-UP, Faculty of Sports and CIFI2D, The University of Porto, 4200-450 Porto, Portugal

**Keywords:** dual-task, EMG, fNIRS, muscle activity, prefrontal cortex, co-contraction index

## Abstract

Postural control depends on attentional resources besides automatic processes. The dual-task paradigm is a possible approach to analyzing the interference and performance between motor and/or cognitive tasks. Various studies showed that, when individuals simultaneously perform two tasks, the postural stability can decline during a dual-task compared with a single-task due to the attentional resources required performing the tasks. However, little is known about the cortical and muscular activity pattern during dual-task performance. Therefore, this study aims to analyze the muscular and prefrontal activity under dual-task performance in healthy young adults. Thirty-four healthy young adults (mean age ± SD = 22.74 ± 3.74 years) were recruited to perform a postural task (standing posture) and a dual-task (maintaining standing posture while performing a cognitive task). Lower-limb muscle activity was bilaterally collected from five muscles using surface electromyography (sEMG), and the co-contraction index (CCI) was also calculated for selected muscle pairings. The oxy- and deoxyhemoglobin concentrations (prefrontal cortex activity) were recorded using functional near-infrared spectroscopy (fNIRS). Data were compared between single- and dual-task performance. Prefrontal activity increased (*p* < 0.05), and muscle activity decreased in most analyzed muscles (*p* < 0.05), from the single-task to cognitive dual-task performing. The co-contraction index patterns changed from single- to dual-task conditions in most selected muscle pairs (*p* < 0.05). We conclude that the cognitive task negatively interfered with motor performance once the muscle activity decreased and the prefrontal cortex activity increased under a dual-task, suggesting that young adults prioritized cognitive task performance, and they allocated more attentional resources to the cognitive task over the motor performance. Understanding the neuromotor changes can help adopt a better clinical practice to prevent injuries. However, future studies are recommended to assess and monitor muscular and cortical activity during the dual-task performance to provide additional information about the cortical and muscular activity patterns in postural control while performing a dual-task.

## 1. Introduction

The literature reports an interaction between some cognitive functions and motor performance/function in healthy and diseased individuals [1,2,3]. An approach to assess that influence is the dual-task paradigm. In real-life situations, when individuals perform their daily tasks or respond to unexpected situations, they adopt strategies to maintain or recover adequate postural control. However, when they simultaneously perform various tasks, the performance of one or both tasks can decrease due to the attentional resources required to perform the tasks [4]. Thus, the loss of balance can happen due to the brain center’s inability to adequately allocate the attentional resources necessary for postural stability [5].

Most studies included in a systematic review [6] assessed the effect of a dual-task on postural control through the postural sway analysis. They reported impairments in postural stability during dual-task performance in neurological conditions and healthy individuals. Furthermore, the results obtained in young adults were ambiguous; some studies showed enhancements in postural stability, others a decrease.

Postural control is a complex motor skill resulting from the interaction of multiple sensorimotor processes and neuro-musculoskeletal systems [7]. Therefore, the human standing posture also depends on the balance between the load stiffness at the ankle resulting from gravity and the ankle stiffness created by ankle muscle and tendon structures [8]. In addition, muscle co-contraction is important for joint stabilization during the motor performance, and it defines the simultaneous contraction of agonist and antagonist muscles around a joint [9]. Furthermore, maintaining standing postural control requires attentional resources and the involvement of cortical networks [4,10].

Studies assessing muscle activity and co-contraction during dual-task performance are scarce. However, they showed that lower extremity muscle activity could be altered under dual-task conditions and affect postural control performance [5,11,12]. A study showed that during the cognitive dual-task performance, the elderly reduced their lower-limb muscle activity and increased their postural sway; however, this behavior did not happen in young adults [12]. One of the tools to measure muscle activity is surface electromyography (sEMG). It is a non-invasive technique that measures electrical muscle activity through surface electrodes placed on the skin overlying the muscle fibers [13,14].

On the other hand, studies that assess the cortical activity during a dual-task have been growing in recent years. The prefrontal cortex can play a role in selecting the appropriate motor responses according to various conditions in maintaining balance [15]. Functional near-infrared spectroscopy (fNIRS) is one of the neuroimage techniques used to analyze the prefrontal cortex activity by measuring the hemodynamic responses of neuronal cortical tissues [16]. Some studies reported increased prefrontal cortex activity during dual-task performance [17,18,19,20]; others found a decrease due to the load of the cognitive tasks [21] and a reduction in prefrontal activation during dual-task conditions compared with single-task walking [22]. The results of these studies show different prefrontal cortex activation strategies under dual-task conditions possibly due to the type of task, age, and health condition.

The dual-task paradigm helps determine the capacity of individuals to divide their attention between two tasks and the effect of concurrent tasks on motor performance [23]; it can provide predictive information on the performance of skills in sports [24,25] and the risk of falls [26,27].

Therefore, understanding the functional connectivity of the brain and muscle activity during dual-task performance can be a valuable tool for assessing the neuromotor performance of healthy and diseased individuals. Furthermore, to the best of our knowledge, limited studies have compared the EMG of lower-limb musculature during single- and dual-task results [5,12], and fewer combined EMG analyses and prefrontal cortex activity [15].

Therefore, we want to contribute to clarifying the interference of the cognitive task over motor control, specifically over muscular activity, combining the assessment of the hemodynamic response in the prefrontal cortex in young adults while simultaneously performing a cognitive and motor task (static standing posture). Thus, this study aims to analyze the muscular and prefrontal activity under dual-task performance in healthy young adults. We hypothesized that (1) in young adults, the addition of a cognitive task while performing a static standing posture (cognitive dual-task) decreases their lower-limb muscle activity and increases the hemodynamic response in the prefrontal cortex than performing a single task; and (2) the co-contraction index decreases from the single- to dual-task performing.

## 2. Materials and Methods

### 2.1. Study Design

This study is an observational and cross-sectional type.

The sample size was calculated using G*power software (Franz Faul, Edgar Erdfelder, Axel Buchner, Universität Kiel, Germany, version 3.1.9.6). Based on the study design, to achieve a large effect size (d = 0.80), in an α = 0.05 and a statistical power of 0.95, a minimum of 24 individuals would be needed.

A total of 34 healthy young adults (23 men and 11 women; mean age ± SD = 22.74 ± 3.74 years; mean ± SD: body mass of 74.30 ± 16.26 kg and height of 1.72 ± 0.09 m) without a history of cognitive, physical, vestibular, or mental disorders participated in this study. This study was approved by the Ethics Committee of the Polytechnic Institute of Coimbra (approval number: 27_CEPC2/2019), and all participants gave informed consent to participate.

### 2.2. Tasks Protocol

The muscle activity by sEMG and hemodynamic response in the prefrontal cortex by fNIRS were collected in single and dual tasks. Each participant performed each task for 60 s, with a rest period between each task of 45 s, twice [28].

In the single-task (ST: motor task), the young adults were instructed to naturally stand upright with their feet shoulder-width apart, eyes open, and arms comfortably at their side along the trunk, without the smartphone [29].

The cognitive dual-task (DT) consisted of simultaneously performing the single-task with a cognitive task. With the purpose of maintaining an ecologically valid study, young adults performed the cognitive dual-task using their smartphone and holding it with their preferred hand or both hands. The cognitive task consisted of arithmetic (sum and subtraction calculations) or visual–spatial memory (memorizing three elements present in pictures) tasks displayed on the smartphone screen. Both tasks involved similar cognitive processes [30]. The participants verbalized the answers, and these were recorded during dual-task performing and while sitting on a chair as a baseline task to assess cognitive task performance by the percentage of correct answers.

### 2.3. Prefrontal Cortex Acquisition and Analysis

The fNIR100A-2 device (Biopac System Inc., Goleta, CA, USA) was used to measure the prefrontal cortex activity based on the cortical response hemodynamic. This device measures oxy- and deoxyhemoglobin concentration changes, recording fluctuations in levels of infrared light at 850 and 730 nm wavelengths. It has 16 recording channels with a source-detector separation of 2.5 cm and records at a frequency of 2 Hz.

Cognitive Optical Brain Imaging Studio and fNIRSoft professional (v3.3) (Biopac software) were used for data acquisition and analysis, respectively. Initially, raw data were visually inspected to remove the optodes that did not present a signal. The raw light intensity was filtered with a low-pass finite impulse response (FIR) filter, with an order of 20 Hamming, and a cutoff frequency set at 0.1 Hz to remove high-frequency noise, cardiac, and respiratory cycle effects [31,32,33]. The sliding-window motion artifact rejection algorithm was used to remove some existing motion artifacts [31].

The modified Beer–Lambert law was used to calculate the changes in the oxygenated and deoxygenated hemoglobin concentrations concerning the 10 s local baseline recorded at the beginning of data collection [28]. In addition, we considered the average concentration of oxy- and deoxyhemoglobin obtained during 60 s of performing the tasks for the analysis of the results.

**Figure 1:**
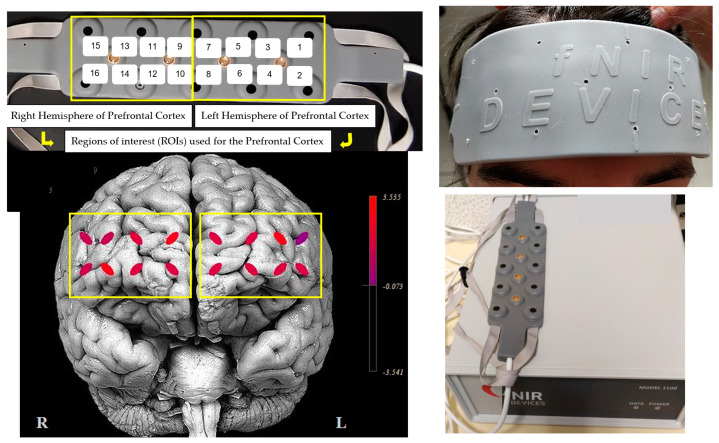
Regions of interest used in prefrontal cortex and fNIR100A-2 device attachment in the participant’s head—device middle mark aligned with the frontal midline.

Considering some regions of interest (ROIs) used for the prefrontal cortex analysis—left dorsolateral prefrontal cortex (channels 1 to 4), left medial prefrontal cortex (channels 5 to 8), right medial prefrontal cortex (channels 9 to 12), and right dorsolateral prefrontal cortex (channels 13 to 16) [34]—we took into consideration for this study that the left hemisphere of the prefrontal cortex includes the mean of channels 1 to 8 (left dorsolateral prefrontal cortex and left medial prefrontal cortex), and the right hemisphere of the prefrontal cortex includes the mean of channels 9 to 12 (right medial prefrontal cortex and right dorsolateral prefrontal cortex) (Figure 1).

### 2.4. Muscular Activity Assessment

**Figure 2:**
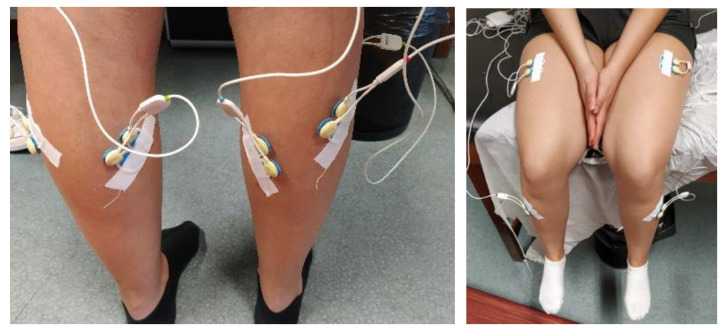
Surface electrode placement in gastrocnemius medialis and lateralis, rectus femoris, and tibialis anterior muscles bilaterally.

Before the electrode placement, the skin was carefully prepared, involving hair removal and cleaning the skin with alcohol to decrease the interface’s impedance between the skin and electrode. Telemetric equipment with Bluetooth connectivity, manufactured by bioPLUX research 2010 (PLUX, Lisbon, Portugal), was used to record and amplify the EMG signals. Active surface electrodes (Al/AgCl, rectangular shape 30 mm × 22 mm) using the AMBU BlueSensor N (AMBU, Ballerup, Denmark) were placed on the left and right sides (Figure 2) of the following muscle bellies: biceps femoris (BF), rectus femoris (RF), tibialis anterior (TA), gastrocnemius medialis (GM), and gastrocnemius lateralis (GL). The electrodes were placed following the descriptions in Hermens et al. [35] and aligned with the muscle fiber orientation with a 20 mm inter-electrode distance at the most prominent part of the muscle bellies. In addition, two ground electrodes were attached to the clavicle bone. EMG signals were amplified with a common-mode rejection ratio of 110 dB and an input impedance superior to 100 mV. Experienced researchers visually inspected the EMG patterns before processing to ensure EMG signal quality. Next, the EMG data were processed using a digital filter at 20–490 Hz, full-wave rectified and smoothed through a low-pass filter at 12 Hz, and processed using a 4th-order Butterworth digital filter with a sampling frequency of 1000 Hz. The maximal voluntary isometric contraction (MVC) was used for amplitude normalization, considering the MVC’s peak 200 ms EMG signal (EMG_MAX_) as a reference. The procedures described by Konrad [36] and Hermens et al. [35] to evaluate maximal voluntary contraction were used. A routine in Matlab software (version R2020b, The Mathworks, Inc., Natick, MA, USA) was used for processing. The EMG average value was calculated during each task performance and participant based on MVC. All participants were verbally encouraged during the maximal isometric efforts, and to avoid fatigue, a 2 min rest period was allowed between repetitions. Three isometric repetitions of 3 to 4 s were performed for each muscle to determine EMG_MAX_.

The co-contraction index (CCI) between the agonist and antagonist muscles—rectus femoris–biceps femoris (RF–BF), tibialis anterior–gastrocnemius lateralis (TA–GL), and tibialis anterior–gastrocnemius medialis (TA–GM) for left and right sides—was calculated using the following Equation (1) [37]:

(1) Co−contraction Index=(lower EMG+higher EMG)∗(lower EMG/higher EMG)

The lower EMG and higher EMG represent the normalized EMG data value (% MVC) of the less active and more active muscle, respectively, at each time point, during single- and dual-task performance.

### 2.5. Statistical Analysis

Statistical normality analysis was assessed for all variables using the Shapiro–Wilk test, showing that the data did not present a normal distribution. Hence, the Wilcoxon test was chosen to assess the differences in the hemodynamic response (concentration of oxy- and deoxyhemoglobin: [oxy-Hb] and [deoxy-Hb], respectively) between ST and DT; to compare the left and right muscle activity and left and right prefrontal activity ([oxy-Hb] and [deoxy-Hb] of ROIs) between single- and dual-task conditions; and to assess the differences in the co-contraction index of left and right muscles between ST and DT. The Kruskal–Wallis test was used to evaluate the differences in the variables under analysis between the hands’ position to hold the smartphone (both hands, right or left hand) once participants performed the DT holding their smartphone with their preferred hand or both hands. Descriptive and EMG data were expressed as mean ± standard deviation (SD). [oxy-Hb] and [deoxy-Hb] were represented in median values (interquartile range (IQR): 25th, 75th percentile). Data were analyzed using IBM SPSS Statistics 25.0 software for Windows (SPSS, Inc., Chicago, IL, USA). The significance level was set at *p* < 0.05 for the present study.

## 3. Results

Young adults performed the cognitive dual-task holding their smartphone with their preferred hand or both hands. However, there were no differences in muscular and prefrontal cortex activity between the young adults holding smartphones with both hands (79.4%) and their preferred hand (left hand: 2.9%; right hand: 17.6%) when performing the cognitive dual-task (*p* > 0.05).

During the single task, there were no differences in muscle activity between the left and right sides of each muscle (TA, GM, GL, RF and BF: *p* > 0.05). However, during the dual-task, differences in muscle activity (% MVC) were found between the left and right sides of each muscle (TA, GM, GL, RF and BF: *p* < 0.05), showing less muscle activity on the right-side muscles compared with the left-side muscles.

**Figure 3:**
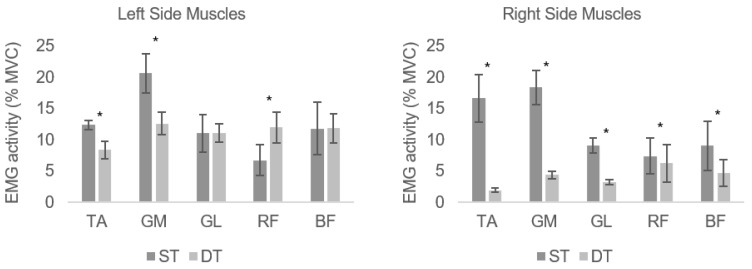
Mean and standard error (error bars) of EMG activity during single- and dual-task. ST, single-task; DT, dual-task; EMG, electromyographic activity (% MVC, maximum voluntary contraction) measured in the TA, tibialis anterior; GM, gastrocnemius medialis; GL, gastrocnemius lateralis; RF, rectus femoris; BF, biceps femoris. * *p*-value < 0.05; Wilcoxon signed test using median values (comparison between ST and DT).

The comparison between single- and dual-task muscle activity (% MVC) is presented in Figure 3. Muscle activity significantly decreased from the single task to the dual-task in the tibialis anterior and gastrocnemius medialis of both sides (left TA: *p* = 0.001 and right TA: *p* < 0.001; left GM: *p* = 0.001 and right GM: *p* < 0.001), gastrocnemius lateralis (*p* < 0.001), rectus femoris (*p* < 0.001), and biceps femoris (*p* < 0.001) of the right side. The left rectus femoris activity was significantly higher during the dual-task performance compared with the single task (*p* = 0.012).

There were no differences (*p* > 0.05) between the single and dual tasks in the left gastrocnemius lateralis and left biceps femoris activity.

There were no differences in the co-contraction index between the left and right sides during the single task (*p* > 0.05). However, the right side’s TA–GM, TA–GL, and RF–BF co-contraction index was lower than the left side’s TA–GM, TA–GL, and RF–BF co-contraction index during the dual-task performance (*p* < 0.05).

**Figure 4:**
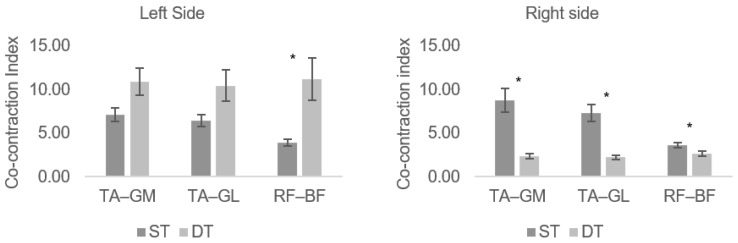
Mean standard error (error bars) of co-contraction index during single and dual tasks. ST, single-task; DT, dual-task; TA–GM, tibialis anterior–gastrocnemius medialis; TA–GL, tibialis anterior–gastrocnemius lateralis; RF–BF, rectus femoris–biceps femoris. * *p*-value < 0.05; Wilcoxon signed test using median values (comparison between ST and DT).

The differences in the co-contraction index between the single and dual tasks are presented in Figure 4. The right side’s TA–GM, TA–GL, and RF–BF co-contraction index was lower during the dual-task performance than that of the single task (*p* < 0.001; *p* < 0.001; *p* = 0.002, respectively). The left RF–BF co-contraction index was higher during the dual-task compared with that of the single task (*p* < 0.001). There was no difference between the single and dual tasks for the left side’s TA–GL and TA–GM co-contraction index (*p* > 0.05).

There was an increase in cognitive task performance, measured by the percentage of correct answers, from the cognitive single task (sitting position) to the dual-task (*p* = 0.007).

In the single- and dual-task performance, no differences were found in the prefrontal cortex’s oxyhemoglobin concentration between the left and right hemispheres (*p* > 0.05). However, the deoxy-Hb concentration was higher in the left prefrontal cortex than in the right prefrontal cortex during the single task (*p* = 0.017); while performing the dual-task, the deoxy-Hb concentration was higher in the right prefrontal cortex than in the left prefrontal cortex (*p* = 0.011).

The changes in hemoglobin concentrations (oxy-Hb and deoxy-Hb) in the prefrontal cortex between the single- and dual-task performance were significant (*p* < 0.05) and are presented in Table 1. The oxy-Hb and deoxy-Hb concentrations increased from the single- to dual-task conditions in the prefrontal cortex region, left hemisphere of the prefrontal cortex, and right hemisphere of the prefrontal cortex.

## 4. Discussion

This study aimed to determine and compare the muscle activity of the lower limbs and the hemodynamic response of the prefrontal cortex when simultaneously performing static standing posture and cognitive tasks in young adults. We hypothesized that muscle activity decreases under dual-task conditions, and the hemodynamic response increases in the prefrontal cortex. Our findings supported this hypothesis by demonstrating that muscle activity decreased from the single task to cognitive dual-task performing in most analyzed muscles, such as tibialis anterior and gastrocnemius medialis of both sides, gastrocnemius lateralis, rectus femoris, and biceps femoris of the right side. On the other hand, the brain activity increased from the single task to cognitive dual-task in the prefrontal cortex, increasing the oxyhemoglobin concentration. These data suggest that the cognitive task negatively interferes with motor performance once the muscle activity decreases during the cognitive dual-task performance in young adults compared with the single task. Furthermore, young adults prioritized cognitive performance over motor performance defined by muscle activity because there was an improvement in cognitive task performance and a decline in motor task performance during the dual-task relative to the single task, suggesting a cognitive-priority tradeoff [38]. The apparent focus on the cognitive task can be reflected in the increase in the prefrontal cortex activity from the single to dual tasks, indicating a higher allocation of attentional resources to cognitive task performance. Beyond that, the decrease in muscle activity in most muscles analyzed from the single to dual tasks can demonstrate muscle relaxation possibly due to the decentralization of attention.

Young adults performed a cognitive task based on the mental tracking/working memory task category [30]. The prefrontal cortex has a role in cognitive control, attention, executive function, and working memory [39]. Furthermore, studies showed a functional connectivity between the dorsolateral prefrontal cortex and primary motor cortex (M1) [40,41]. Concerning our results, we assume that the increase in prefrontal cortex activity during the dual-task performance can contribute to the reduction of efferent motor information and decreased muscle unit recruitment, decreasing the muscular activity of most muscles from the single to dual tasks. Thus, postural control can be compromised, leading to a decline in postural stability. Some studies suggested that a higher muscle co-contraction can be considered a strategy to stiffen the joint and improve postural stability [42,43].

Some similar studies to ours corroborate our results [5,15], but others do not [12]. For example, a study that assessed the influence of ankle muscle activities, coactivation, and dorsolateral prefrontal cortex activity on postural stability during the dual-task showed a higher tibialis anterior muscle activity and tibialis anterior–gastrocnemius lateralis coactivation in the shorter sway path length group than the longer group. Furthermore, the left dorsolateral prefrontal cortex activity was superior during the performance of the dual-task (performing calculations while standing still) than the single task [15]. Another study showed a decline in gastrocnemius and tibialis anterior muscle activity during the cognitive dual-task in young and older adults, suggesting a less attentional processing capacity available to the balance control under dual-task conditions [5]. On the other hand, a study showed that the right leg’s medial gastrocnemius and tibialis anterior activity decreased during cognitive dual-task performing compared with that during the single task (standing in the Romberg stance on a compliant foam surface and holding a glass in the left hand) in older adults; however, no difference in muscle activity was found in young adults [12].

The muscle activity on each right side muscle and the TA–GM, TA–GL, and RF–BF co-contraction index were inferior compared with those of the left side muscles under dual-task conditions. The left prefrontal cortex is associated with working memory, logical process, and speech [44,45,46]. Therefore, higher left prefrontal cortex activity than the right prefrontal cortex can happen because the participants verbalized the answers when performing the cognitive task, contributing to increased load in the left prefrontal cortex. Therefore, the increased hemodynamic response in the left prefrontal cortex during the dual-task can explain the decrease in the right-side muscle activity and co-contraction index. Another reason to explain the difference between the left and right co-contraction index may be due to the postural control strategies adopted during the dual-task to maintain balance, such as the ankle, hip, or mixed strategies [47].

Performing cognitive tasks can reduce central nervous system resources that are utilized during physical tasks requiring maximal voluntary muscular force production [48]. That can explain the decrease in the right lower-limb co-contraction index when young adults performed the cognitive dual-task. Although we investigated a simple motor task (static standing posture), the increased left prefrontal cortex activity from the single to cognitive dual tasks suggests that young adults allocate fewer cognitive resources to motor tasks due to cognitive task effort.

Our results identified differences between muscular and cortical activity in dual-task conditions compared with the single task in healthy young adults. A study that analyzed cortico-muscular coherence after balance perturbations found a higher coherence between cortical activity from the motor cortex (C1—central area) with electromyographic recordings of rectus femoris muscle in elderly and tibialis anterior in young adults [49]. Given these and our results, it would be necessary to combine fNIRS, EEG, and EMG in future research to assess the interactions between motor and cognitive cortical activity with muscular activity during the dual-task performance to add tools to the analysis of the mechanisms involved in motor control. Furthermore, fNIRS can also be used to investigate muscle physiology, evaluating oxidative skeletal muscle performance [50]. Thus, we recommend a future study that evaluates the oxidative skeletal muscle performance and hemodynamic response in the prefrontal cortex using the fNIRS device during dual-task conditions to improve sports performance and the type of training.

We report the following limitations in our study: the fNIRS device that we used only allowed us to measure the prefrontal cortex activity; we did not measure the motor cortex activity. Therefore, in our study, it was not possible to assess the interaction between the prefrontal cortex and brain motor–network areas, such as the motor cortex, limiting the understanding of the motor response resulting from the cognitive task interference during the dual-task performance. In addition, we assume that the reduction in muscle activity and increase in prefrontal cortex activity can compromise postural control by reducing postural stability.

Our research showed that adding a cognitive task while performing a motor task interferes with muscle and prefrontal cortex activity, which can compromise the maintenance of postural control, and might contribute to the risk of falls or musculoskeletal injuries (e.g., the loss of static postural control can occur when being shoved while waiting for the bus in the upright posture). Motor performance is further compromised by the addition of cognitive tasks in individuals with musculoskeletal injuries [51]; therefore, understanding the neurophysiological changes can help adopt the better clinical practice in the rehabilitation and prevention of injuries. In addition, dual-task training can improve the neural network connections between motor and cognitive brain regions and consequently improve dual-task performance [52].

## 5. Conclusions

The muscle activity decreased and the prefrontal cortex activity increased during the cognitive dual-task compared with the single task, suggesting that young adults allocated more attentional resources to the cognitive task over the motor task under dual-task conditions. Furthermore, performing a dual-task can alter co-contraction index patterns in the lower-limb musculature, showing the interference of cognitive tasks over muscle activity. However, future studies are recommended to assess and monitor muscular and cortical activity during the dual-task performance to provide additional information about the cortical and muscular activity patterns in postural control while performing a dual-task.

## Figures and Tables

**Table 1:** Comparisons of hemodynamic response among single task and cognitive dual-task, median (IQR).

		Single-Task	Dual-Task	*p*-Value ^1^
[oxy-Hb]	PFC	0.419 (−0.099–0.660)	0.812 (0.025–1.297)	0.029
	LPFC	0.393 (−0.132–0.755)	0.689 (−0.193–1.489)	0.033
	RPFC	0.302 (−0.107–0.677)	0.525 (0.154–1.437)	0.035
[deoxy-Hb]	PFC	−1.864 (−2.916–(−1.239))	−0.897 (−2.347–0.530)	0.001
	LPFC	−1.614 (−2.903–(−0.984))	−0.909 (−2.601–(−0.289))	0.008
	RPFC	−1.974 (−2.891–(−1.166))	−0.883 (−2.634–0.938)	0.001

[oxy-Hb], oxyhemoglobin concentration (μmol/L); [deoxy-Hb], deoxyhemoglobin concentration (μmol/L); PFC, prefrontal cortex; LPFC, left prefrontal cortex; RPFC, right prefrontal cortex. ^1^ Wilcoxon signed test.

## Data Availability

Not applicable.
